# Colic incidence, risk factors, and therapeutic management in a working horse population in Tuban, Indonesia

**DOI:** 10.14202/vetworld.2024.963-972

**Published:** 2024-05-04

**Authors:** Faisal Fikri, Dodit Hendrawan, Arya Pradana Wicaksono, Agus Purnomo, Shafia Khairani, Shekhar Chhetri, Muhammad Thohawi Elziyad Purnama, Hakan Çalışkan

**Affiliations:** 1 Department of Veterinary Science, Faculty of Health, Medicine, and Life Sciences, Universitas Airlangga, Banyuwangi, Indonesia; 2 Animal Health Division, Indonesian Horse Veterinarian Association, Surabaya, Indonesia; 3 Department of Veterinary Surgery and Radiology, Faculty of Veterinary Medicine, Universitas Gadjah Mada, Yogyakarta, Indonesia; 4 Department of Biomedical Science, Faculty of Medicine, Universitas Padjajaran, Bandung, Indonesia; 5 Department of Animal Science, College of Natural Resources, Royal University of Bhutan, Lobesa, Punakha, Bhutan; 6 Department of Biology, Graduate School of Natural and Applied Sciences, Eskişehir Osmangazi Üniversitesi, Eskişehir, Türkiye; 7 Department of Biology, Faculty of Science, Eskişehir Osmangazi Üniversitesi, Eskişehir, Türkiye

**Keywords:** colic, domesticated animals, horse, risk factors, therapeutic management

## Abstract

**Background and Aim:**

Colic is the primary problem affecting equestrian care worldwide. The primary cause of colic is digestive diseases; however, they can also affect organs from different systems in the abdominal region. In addition to a prior history of the disease and its treatment, risk factors may be assessed to determine the etiology of the disease in horses without or with a history of colic. This study aimed to present a summary of the incidence, risk factors, and medical procedures for colic in horses.

**Materials and Methods:**

Based on owner reports, 223 horses in Tuban, Indonesia, suspected of having colic were investigated. During the investigation of clinical parameters, investigators went door-to-door with interested horse owners to gather information about potential risk factors related to equine colic. Information on horses diagnosed with colic was obtained from the medical records of treatment. A Chi-square test was used to investigate the potential association between the risk factors, medical protocol, and the outcome of colic in horses.

**Results:**

Of the 187 cases, spasmodic colic was the most common (48.13%), but 17 (9.09%) had no definitive diagnosis. Poor body condition scores (χ^2^ = 58.73; p < 0.001), wheat bran feeding (χ^2^ = 26.79; p < 0.001), concentrate (χ^2^ = 10.66; p < 0.01), less access to water (χ^2^ = 128.24; p < 0.001), recurrence of colic (χ^2^ = 85.64; p < 0.001), no deworming program (χ^2^ = 54.76; p < 0.001), the presence of gastrointestinal parasites (χ^2^ = 56.79; p < 0.001), stressed physical activity (χ^2^ = 28.53; p < 0.001), and summer season (χ^2^ = 7.83; p < 0.01) were the risk factors for colic. We further reported that 185 (98.93%) patients who received the following medical interventions recovered: injection of non-steroidal anti-inflammatory drugs was necessary, Vitamin B complex (χ^2^ = 39.98; p < 0.001), fluid therapy (χ^2^ = 92.99; p < 0.001), and gastric intubation (χ^2^ = 4.09; p < 0.05).

**Conclusion:**

The importance of colic was demonstrated in 187 (83.86%) of the 223 horses investigated in Tuban, Indonesia, documented. In this study, recommendations for medical procedures when colic risk factors have been determined are presented.

## Introduction

Abdominal pain is one of the most common causes of critical diagnoses in equine veterinary treatment, which contributes to about 30% of emergency calls to equine practitioners [1]. Horses suffering from colic can have various causes, but the most prevalent is acute gastrointestinal illness that affects the abdominal organs [2]. According to a number of international studies, colic is the most frequent cause of emergency veterinary care and the leading cause of death or euthanasia. A moderate incidence of colic has been documented in Ireland (38.4%) [3], Sweden (50.0%) [4], Netherlands (53.4%) [5], Greece (29.8%) [6], UK (33.3%) [7], USA (5.6%) [8], Denmark (56.4%) [9], Nigeria (51.4%) [10], and Egypt (55.8%) [11]. In Albania [12] and Iran [13], high incidence levels (100%) have been reported.

The term “colic” refers to a wide range of disorders affecting the abdominal organs because it has vastly different etiologies and a common feature of abdominal pain originating in the digestive tract. Although all of these diseases have slightly distinctive symptoms that could lead to a probable or even positive diagnosis, it is partly important to converge them under one term because many diseases cannot be identified during life [14]. The majority of colic cases are caused by the careless handling of horses by owners and can easily be prevented with appropriate caution and judgment. For all intents and purposes, three distinct colic-related diseases were identified: Spasmodic colic, flatulent colic, and inflammation of the intestine or stomach. These three disease types exhibit a shared set of symptoms as well as additional signs that are exclusive to each specific form and hence diagnostic [15]. In general, a prolonged fasting period, during which the animal is likely to swallow food without properly preparing it for stomach and intestinal digestion, is one of the main causes of colic. Other causes include incorrect feeding, sudden changes in diet, and excessive feeding. Other conditions that may occasionally cause colic include intestinal concretions, mesenteric abscesses, and parasites [16]. In previous epidemiological studies, there have been variables linked to a change in the risk of colic. The season is one of them, together with several management and risk factors specific to horses, such as age, gender, breed, wind-sucking habits, parasites, nutritional status and feeding practices, physical activity, access to housing and pasture, availability of water, movement, and vaccination. According to these results, colic is a complex condition, and recurrent colic is more likely to occur in horses who have already experienced one episode [17].

There is currently limited information on horses with a higher risk of colic and risk factors at management level. In this study, horses with colic and horses without colic or with a history of colic were compared. This study evaluates the efficacy of treatment during colic episodes and the likelihood of recurrence of colic and presents a recap of the incidence, risk factors, and therapies associated with colic in Tuban.

## Materials and Methods

### Ethical approval

In this study, horse owners provided written cooperative consent to investigate management factors associated with colic. However, during the examination of clinical parameters, investigations, diagnosis, and medical treatment, the standard operational protocols of the Indonesian Horse Veterinary Association should be followed. The ARRIVE guidelines to improve the design, analysis, and publication of animal research (10.6084/m9.figshare.25563222) were considered in this study.

### Study period and location

This investigation was conducted from August 2021 to March 2023 in Tuban, Indonesia, which is geographically located at 6°53’41.2”S 112°03’56.0”E. All reports from horse owners pertaining to colic were addressed, and every depiction of horses and herds in a single stable or nearby location was assessed.

### Study design and sample collection

A prospective study designed to enroll 223 horses with abdominal pain in Tuban, Indonesia, was evaluated based on owner reports. During the clinical parameters examination, investigators visited willing horse owners to collect questionnaires about risk factors that may be related to equine colic. Equine veterinarians who provided the first opinions served as investigators in the diagnosis of probable colic cases in horses, and the horses were treated medically. This study excluded horses who had previously undergone colic surgery, were younger than 6 months of age, pregnant, had foals at the foot, or experienced abdominal pain due to non-gastrointestinal causes. Horses admitted multiple times in 12 months were evaluated only for the initial admission to assess risk factors.

The medical report must document the dates of colic episodes, specific clinical parameters in the abdominal region, heart rate (HR), breathing rate, mucous appearance, signs of dehydration, bowel movements, appetite, perspiration, urination, body temperature, and colic-type classification. Regardless of the outcome or severity of the case, the owners of horses were requested to provide information for all horses referred for a colic investigation. Four components, each with a variety of question formats, were included in the questionnaire designed for colic risk factor assessment forms: intrinsic factors, feed management, medical history, and environmental factors. Risk factors that were retrieved from in-depth interview analysis were: age (<5 years/5−10 years/>10 years), gender (male/female), breed (Sandalwood/Bima/Thoroughbred/Mixed), body condition score (poor/good), wheat bran feeding (none/yes), green fodders (none/yes), whole corn (none/yes), fruit or vegetables (none/yes), concentrate (none/<5 kg/>5 kg), probiotics (none/yes), water source (soft/well), access to water (once/twice/three/more than 3 times), recurrence of colic (absent/present), deworming (none/yes), gastrointestinal parasites (absent/present), dental diseases (absent/present), musculoskeletal diseases (absent/present), activity (exercise/intense/stressing/changes in activity), season (summer/winter), and housing system (indoor stalling/changes in housing).

For those horses diagnosed with colic, the following data were retrieved from medical treatment records: gastric intubation (none/yes), Vitamin B complex (none/yes), Nonsteroidal anti-inflammatory drugs (NSAIDs) (none/yes), analgesics (none/yes), fluid therapy (none/yes), spasmolytics (none/yes), and antibiotics (none/yes). The telephone enquiry questionnaire recorded any additional colic episodes and noted data that could change during the investigation period. Behavior-related data were not collected during follow-up because it was thought that behavior would not change much for the study. The investigators asked the participating owners to inform them of any recurring episodes of colic in their horses for further investigations. They also asked whether the recurrence complied with the case definition, provided an inventory of the colic, and communicated treatment management information.

### Horse treatment

As soon as the clinical characteristics were documented in the medical report and determined to be one of the colic types, the medical team promptly started treatment. In most cases, jugular vein intravenous fluid therapy was administered at least 5–10 L or 10–20 flacons of Lactated Ringers’ solution (Otsu RL^®^, Otsuka, Indonesia). One of the NSAIDs was administered intravenously during treatment and monitored concurrently. The following NSAID types were utilized: Flunixin meglumine (Flumine^®^, Jaapharm, Mano, Singapore), phenylbutazone (Phenylbute^®^, Phoenix Pharm, USA), and ketoprofen (Ketofen^®^, Zoetis, USA) were routinely injected at a dosage of 1 mg/kg intravenously q 12 h, 2.2–4.4 mg/kg intravenously q 12–24 h, or 2.2 mg/kg intravenously q 24 h, respectively. Spasmolytic (5 mL/100 kg intravenously; Buscopan Compositum^®^, Boehringer Ingelheim, Germany) or opioid (1 mg/kg intravenously; Torbugesic-SA^®^, Fort Dodge, USA) injection was combined with NSAIDs to induce a weak visceral analgesic in some cases. Vitamin B complex (B-Sanplex^®^, Sanbe, Indonesia) was intramuscularly injected at a dosage of 10 mL/200 kg to induce energy metabolism [18]. We also utilized a double-lumen nasogastric tube with a larger diameter (7.8 mm input diameter and 12.5 mm output diameter × 2.7 m) in our study. A double-lumen nasogastric tube lubricated with sterile jelly was carefully inserted into the stomach through the right nostril while taking into account the tracheal pressure. Gastric intubation was performed to deliver water and mineral oil into the horses’ stomach as a laxative. The main basis for rigorous medical treatment was the severity of clinical parameters and ongoing monitoring throughout the case assessment and recovery phases [19].

### Statistical analysis

All data were compiled and archived in an Excel spreadsheet (Microsoft Office 2013, version 13.0) for data clarification and rechecked for correctness by the primary researcher. Clinical parameters, diagnosis, and pain management in horses with colic were tabulated and reported as frequency counts (percentage of the total incidence). Data were extracted and recategorized using defined nominal data to assess the effect of risk factors that were investigated during the study. This included intrinsic factors, feed management, medical history, and environmental factors between each of the follow-up questionnaires for both normal and colic-associated horses. To evaluate the efficacy of therapeutic management, only colic-associated horses were included in the analysis.

A Chi-square (χ^2^) test was first used for all horses in the study and then only for horses diagnosed with colic to determine if there was a potential link between probable risk variables and the outcome. In addition, odds ratio, relative risk, and 95% confidence interval (95%) were presented to emphasize the interpretation of risk outcomes. At p < 0.05, the factors were considered to be significantly linked with the results. Statistical Package for the Social Sciences v.25 Software (IBM Corporation, Armonk, NY, USA) was used for all analyses.

## Results

A total of 187 cases (83.86%) of 223 samples were reported in horses with colic episodes. The HR >80 beats/min (57.75%), abnormal breath rate (54.01%), abnormal mucous membrane color (51.34%), moistness (67.38%), moderate dehydration (51.87%), profuse sweating (96.26%), frequent urination (81.82%), and elevated rectal temperature (95.19%) were observed in most of these horses. We also reported severe typical abdominal pain signs such as flehmen (88.77%), kicking at the belly (80.75%), flank watching (79.14%), and pawing at the ground (79.14%). On the basis of general GI evaluation, constipation (61.50%), intestinal sound (78.07%), and anorexia (80.75%) were observed in these horses (Table-1).

**Table 1:** Clinical parameters in 187 horses referred with signs of colic.

Clinical parameters	Colic (n = 187)	Percentage (%) of horses
Heart rate		
<80 beats/min	79	42.25
>80 beats/min	108	57.75
Respiratory rate		
Normal rate 8−16 breaths/min	86	45.99
Abnormal	101	54.01
Mucous membrane colour		
Normal colour	91	48.66
Abnormal	96	51.34
Mucous membrane hydration		
Normal moist	61	32.62
Abnormal	126	67.38
Dehydration		
Mild	77	41.18
Moderate	97	51.87
Severe	13	6.95
Abdominal pain		
Flehmen	166	88.77
Kicking at the belly	151	80.75
Flank watching	148	79.14
Pawing at the ground	148	79.14
Rolling	21	11.23
Abdominal distention		
Absent	172	91.98
Present	15	8.02
Intestinal movement		
Absent	23	12.30
Constipation	115	61.50
Diarrhea	49	26.20
Intestinal sound		
Absent	41	21.93
Present	146	78.07
Appetite		
Anorexia	151	80.75
Good	36	19.25
Profuse sweating	180	96.26
Frequent urination	153	81.82
Congested mucous membrane	137	73.26
Elevated rectal temperature	178	95.19

n=Number of samples

Diagnostic aids were used to determine the type of colic and therapies were implemented to treat horses during colic periods. As shown in Table-2, spasmodic colic was indicated in 90 cases (48.13%) of horses, but no definitive diagnosis was made in 17 cases (9.09%) due to the complexity of clinical parameters and individual horse circumstances. We used a combination of NSAIDs + spasmolytics (44.92%) and NSAIDs + opioids (44.39%) in several cases, with flunixin meglumine (93.05%) being the most commonly used NSAID.

**Table 2:** Diagnosis and pain management for 187 horses referred for colic treatment.

Treatments	Number of horses	Percentage (%) of horses
Diagnosis (n = 187)		
Impaction colic	22	11.76
Spasmodic colic	90	48.13
Strangulation	2	1.07
Enteritis or colitis	45	24.06
Intestinal displacement	11	5.88
No definitive diagnosis	17	9.09
Pain management (n = 187)		
NSAIDs	13	6.95
NSAIDs, Spasmolytics	84	44.92
NSAIDs, Opioids	83	44.39
NSAIDs, alpha-2-agonists	7	3.74
NSAIDs type (n = 187)		
Flunixin meglumine	174	93.05
Phenylbutazone	8	4.28
Ketoprofen	5	2.67

n=Number of samples, NSAIDs=Nonsteroidal anti-inflammatory drugs

The main purpose of this study was to identify factors that play a crucial role in promoting the risk of colic in horses. Poor body condition scores significantly contributed to colic (χ^2^ = 58.73; p = 0.001) based on intrinsic factors (Table-3). Meanwhile, we evaluated feed management factors and reported an increased risk of colic-associated with wheat bran feeding (χ^2^ = 26.79; p < 0.001), concentrate (χ^2^ = 10.66; p < 0.01), and less access to water (χ^2^ = 128.24, p < 0.001). The addition of fruit, vegetables, and probiotics is not recommended in the ideal horse diet (Table-4).

**Table 3:** Chi-square model of intrinsic factors associated with colic in horses population in Tuban.

Variables	Normal (n = 36)	Colic (n = 187)	χ^2^	p-value	OR	RR	95% CI
Age							
<5 years	12	41	3.59	0.166	n/a	n/a	n/a
5−10 years	18	90				n/a	n/a
>10 years	6	56				n/a	n/a
Gender							
Male	18	96	0.02	0.883	0.95	0.97	0.68−1.39
Female	18	91				1.03	0.72−1.47
Breed							
Sandalwood	13	96	7.16	0.067	n/a	n/a	n/a
Bima	16	43				n/a	n/a
Thoroughbred	5	34				n/a	n/a
Mixed	2	14				n/a	n/a
Body condition score							
Poor	4	144	58.73	0.000***	0.04	0.14	0.06−0.37
Good	32	43				3.87	2.90−5.15

Significant at *p < 0.05, **p < 0.01,

*** p < 0.001. n=Number of samples, χ^2^=Chi-square, OR=Odds ratio, RR=Relative risk, 95% CI=95% confidence interval, n/a=not applicable

**Table 4:** Chi-square model of feed management factors associated with colic in horses population in Tuban.

Variables	Normal (n = 36)	Colic (n = 187)	χ^2^	p-value	OR	RR	95% CI
Wheat bran feeding							
None	2	98	26.79	0.000***	0.05	0.11	0.03−0.41
Yes	34	89				1.98	1.67−2.35
Feeding on green fodders							
None	26	147	0.71	0.400	0.71	0.92	0.74−1.14
Yes	10	40				1.29	0.72−2.35
Whole corn in diet							
None	33	183	3.81	0.051	0.24	0.94	0.85−1.04
Yes	3	4				3.89	0.91−16.67
Fruit or vegetables in diet							
None	35	184	0.24	0.627	0.57	0.99	0.93−1.05
Yes	1	3				1.73	0.19−16.18
Concentrate feeding							
None	3	16	10.66	0.005**	n/a	n/a	n/a
<5 kg	4	72				n/a	n/a
>5 kg	29	99				n/a	n/a
Probiotics in diet							
None	36	185	0.39	0.533	n/a	1.02	0.99−1.03
Yes	0	2				n/a	n/a
Water source							
Soft water	20	92	0.49	0.485	1.29	1.13	0.82−1.57
Well	16	95				0.88	0.59−1.29
Access to water/day							
Once	0	48	128.24	0.000***	n/a	n/a	n/a
Twice	1	120				n/a	n/a
3 times	23	16				n/a	n/a
More than 3 times	12	3				n/a	n/a

Significant at *p < 0.05,

** p < 0.01,

*** p < 0.001. n=Number of samples, χ^2^=Chi-square, OR=Odds ratio, RR=Relative risk, 95% CI=95% confidence interval, n/a=Not applicable

Colic is also related to the horse’s medical history. In the present study, we highlighted the contribution of colic recurrence (χ^2^ = 85.64; p < 0.001), no deworming program (χ^2^ = 54.76; p = 0.001), presence of gastrointestinal parasites (χ^2^ = 56.79; p < 0.001), and stressed physical activity (χ^2^ = 28.53; p < 0.001) on colic episodes (Table-5). As shown in Table-6, the summer season (χ^2^ = 7.83; p < 0.01) was reported to be an environmental factor that increased the risk of colic.

**Table 5:** Chi-square model of medical history factors associated with colic in horses population in Tuban.

Variables	Normal (n = 36)	Colic (n = 187)	χ^2^	p-value	OR	RR	95% CI
Recurrence of colic							
Absent	28	18	85.64	0.000***	32.86	8.08	5.04−12.96
Present	8	169				0.25	0.13−0.45
Deworming program							
None	0	125	54.76	0.000***	n/a	n/a	n/a
Yes	36	62				3.02	2.46−3.69
Gastrointestinal parasites							
Absent	36	60	56.79	0.000***	n/a	3.12	2.53−3.84
Present	0	127				n/a	n/a
Dental diseases							
Absent	34	173	1.17	0.681	1.38	1.02	0.93−1.12
Present	2	14				0.74	0.18−3.13
Musculoskeletal diseases							
Absent	36	171	3.32	0.069	n/a	1.09	1.05−1.14
Present	0	16				n/a	n/a
Activity							
Exercise	29	62	28.53	0.000***	n/a	n/a	n/a
Intense	4	52				n/a	n/a
Stressing	2	62				n/a	n/a
Changes in activity	1	11				n/a	n/a

Significant at *p < 0.05, **p < 0.01,

*** p < 0.001. n=Number of samples, χ^2^=Chi-square, OR=Odds ratio, RR=Relative risk, 95% CI=95% confidence interval, n/a=not applicable

**Table 6:** Chi-square model of environmental factors associated with colic in horses population in Tuban.

Variables	Normal (n = 36)	Colic (n = 187)	χ^2^	p-value	OR	RR	95% CI
Season							
Summer	17	133	7.83	0.005**	0.36	0.67	0.47−0.95
Winter	19	54				1.83	1.25−2.68
Housing							
Indoor stalling	36	181	1.19	0.276	n/a	1.03	1.01−1.06
Changes in housing	0	6				n/a	n/a

Significant at *p < 0.05,

** p < 0.01, ***p < 0.001. n=Number of samples, χ^2^=Chi-square, OR=Odds ratio, RR=Relative risk, 95% CI=95% confidence interval, n/a=Not applicable

Among the 187 horses with colic, 98.9% (n = 185) were recovered and 1.1% (n = 2) died. Horses with colic were therapeutically treated with NSAIDs either individually or in combination with the prescribed treatment regimen. Gastric intubation (χ^2^ = 4.09, p < 0.05) was performed to decompress the GI tract. Horse laxatives were delivered through a nasogastric tube in the form of mineral oil and water. Fluid therapy (χ^2^ = 92.99; p < 0.001) and Vitamin B complex (χ^2^ = 39.98; p < 0.001) were administered to hydrate, promote energy metabolism, and partially alleviate the production of lactic acid (Table-7).

**Table 7:** Therapeutic management associated with colic in recovered (n = 185) and dead (n = 2) horses.

Treatments	Recovered (n = 185)	Died (n = 2)	χ^2^	p-value	OR	RR	95% CI
Gastric intubation							
None	60	2	4.09	0.044*	n/a	0.32	0.26−0.39
Yes	125	0				n/a	n/a
Administration of Vitamin B complex							
None	7	2	39.98	0.000***	n/a	0.04	0.02−0.08
Yes	178	0				n/a	n/a
Administration of NSAIDs							
None	0	0	n/a	n/a	n/a	n/a	n/a
Yes	185	2				n/a	n/a
Combination of analgesics							
None	96	1	0.00	0.958	1.08	1.04	0.26−4.18
Yes	89	1				0.96	0.24−3.88
Fluid therapy							
None	0	1	92.99	0.000***	n/a	n/a	n/a
Yes	185	1				2.00	0.50−7.99
Administration of spasmolytics							
None	102	1	0.02	0.885	1.23	1.10	0.27−4.44
Yes	83	1				0.89	0.22−3.62
Administration of antibiotics							
None	110	2	1.35	0.245	n/a	0.59	0.53−0.67
Yes	75	0				n/a	n/a

Significant at

* p < 0.05, **p < 0.01,

*** p < 0.001. n=Number of samples, χ^2^=Chi-square, OR=Odds ratio, RR=Relative risk, 95% CI=95% confidence interval, n/a=not applicable

## Discussion

To support the diagnosis of colic, it is essential to monitor clinical indicators immediately after the symptoms of abdominal pain are noticed. The previous studies by Bowden *et al*. [1] and Purnama *et al*. [16] have also demonstrated that colic symptoms might be accompanied by symptoms such as pale and congestion-ridden mucous membranes, severe dehydration, bowel movement, loss of eating habits, rapid heartbeat per minute, abundant sweat, excessive urine, and rising body temperature. HR, stomach reflux, packed cell volume, capillary refill time, and color of mucous membranes—which can range from cyanotic to brick red—have all been linked favorably in a number of cases involving horses diagnosed with colic [17].

There are a number of potential causes of equine colic; however, only a small number of risk variables have sufficient evidence to support this. A retrospective analysis of the medical records of horses that were referred for colic syndrome was conducted to determine the prognostic and risk factors [20]. Majority of cases in this study (48.13%, n = 90) were spasmodic colic. Spasmodic colic is often accompanied by abdominal discomfort, which occurs frequently but rarely persists. Intestinal spasms and increased peristalsis compress the nerves, causing abdominal pain. Diarrhea is another symptom of increased peristalsis. Spasmodic colic can be caused by indigestible food or sudden change of diet [21]. The symptoms of impacted colic include constipation, sadness, and mild abdominal pain. Lack of food, lack of access to clean water, exhaustion, dental disease, illness, or surgery are the causes of this situation. Acute cases of colonic disease typically result from a significant increase in stomach capacity [22]. Another study documented 255 (68%) cases of spasmodic colic in Kenya with the following clinical signs: vomiting, acute or increasing discomfort, excessive anorexia, and restlessness. Lethargy and shock symptoms seem to predominate in a more advanced illness. Invagination, volvulus, and strangulation are examples of alterations in the intestine’s structure that may be the cause of obstruction if they are discovered within the intestine. Obstructive colic pain progresses over time [23].

Because stallions and Sumba horse breeds are often kept in Indonesia, the results of a previous study by Fikri *et al*. [24] indicated their association as risk factors for colic. In contrast, this study reported that age, breed, maturity, and gender have no association with the occurrence of colic in horses. However, the owners of the Sandalwood breed in Indonesia extensively raise them. The Sandalwood horse, domesticated on the island of Sumbawa, is well-known for its remarkable agility. Due to their propensity to locate and thrive in tropical areas, sandalwood horses are often employed as traditional modes of transportation [25]. In another study [26], the risk was higher for breeding horses than for pleasure horses (e.g., Arabian breeds vs. Thoroughbreds), horses under the care of trainers or managers as opposed to owners, horses whose diets included a large amount of maize, and horses kept in outdoor enclosures without access to water.

This study further demonstrated that consumption of feed (e.g., wheat bran, concentrate >5 kg, and limited water access) can raise the incidence of colic. In this study, we identified several interconnected concentrate feeding characteristics that may be changed to lower the risk of colic in horses. The feeding quantity, type, and frequency concentration variables could be assessed separately; however, it was impossible to determine how they interacted with one another [27]. The most explanatory of these factors showed that the incidence of colic was 6 times higher in horses with the highest concentration of concentrate than in pasture-dwelling horses without concentrate. The risk associated with high concentrations of concentrate was not mitigated by feeding a large amount of concentrate in three or more feedings per day [28]. In a previous study, the risk of colic was increased by 4.8 and 6.3 for feeding concentrates weighing 2.5 kg or more daily. The more concentrate ingested, the higher the risk of colic [29]. The likelihood of colic was reduced by 1.7 compared to a horse that was not fed a whole grain diet, such as barley, oats, or another unprocessed grain. Significant risk factors included variations in the amount or type of concentrate feed and more hay than expected in a single year. Therefore, the horse is more vulnerable if the owner makes several dietary changes [30].

In addition, we found no correlation between colic and the inclusion of fruit, vegetables, and probiotics in a horse’s diet. In an earlier investigation, a noteworthy interplay was found in which the heightened risk associated with wind-sucking was altered by the inclusion of fruits and vegetables [31]. However, probiotics may be associated with a higher incidence of recurring colic, which should be investigated properly [32]. The administration of probiotics may serve as a marker for specific types of horses, such as older horses, horses with dental problems, horses at risk of laminitis, or horses with a history of colic. The effect of probiotics on equine colic is not well understood. One study, which was not directly related to ours, found that giving *Lactobacillus pentosus* preparation to foals was linked to colic symptoms and diarrhea [33]. However, there are still unanswered questions about the effectiveness of probiotic supplements, and further clinical studies are needed to explore their potential for treating horses.

The increased risk of colic may be attributed to a horse’s previous medical history. In this study, colic recurrence, lack of deworming program, presence of gastrointestinal parasites, and stressful physical activity were all mentioned. It has been suggested that parasites are a primary cause of colic. Each farm stated that at least some horses used anthelmintics during the year [34]. Worm infestation may result from a single infection or a mixture of them. Treatment of mixed infections is often enhanced by deworming. Variations in the resistance of horses to worm infection lead to different types of worm infestation. Multiple worm infections may be the cause of weakened immune systems in horses. Due to the high proportion of horses who did not use anthelmintics and the strength of other explanatory factors, these groups did not have an influence on the multivariable analysis [35]. In a previous study [36] conducted in Texas, the incidence of colic was 2.2 times higher in horses that did not receive regular deworming treatments. Colic is less likely if there is a deworming program aimed at reducing the number of strongyle eggs per gram of feces to less than 200. However, another study reported that horses prescribed anthelmintic medicine during the previous 6 months had a higher likelihood of having a colic episode within the following year [37]. This result may initially seem illogical in light of previous study that has identified colic risk factors. It is challenging to determine the underlying cause of this connection because anthelmintic medications may be administered in response to colic or colic symptoms associated with parasites [38]. However, as far as equestrian activities are concerned, our assessment indicated that stressful activities during training and competition may have led to colic. The same consequences relate to stress-related activities. In addition, a previous study reported that the probability of colic was 2.2-fold higher in horses who experienced a change in activity within 2 weeks before the evaluation. This effect could also be explained by the corresponding dietary adjustments or stabling [39].

In this study, only seasonal variables affecting the probability of colic were described in light of environmental factors. The summer season can increase the chance of colic by 1.8 times [40]. In a previous study, there was a possible link between colic and weather-related variables. There was no discernible association between temperature and barometer fluctuations in the 24 h leading up to the colic crisis [41]. However, a study found that the incidence of colic increased in summer. The risk of colic has increased by 3.2 in response to changes in weather during the 3 days preceding the investigation. A previous study by Diakakis and Tyrnenopoulou [6] revealed a significant adverse association between regular temperature and humidity levels for temperatures above 10°C. In this study, the potential of horse housing to prevent colic was further assessed. In most cases, the horses in our study were housed in indoor stalls; therefore, there was no significant contribution. The risk of colic appears to be influenced by housing conditions [42]. In comparison with horses kept indoors, horses kept on pastures throughout the year are less likely to suffer colic. Equine colic is mainly caused by changes in the management of housing. Dietary and exercise changes are frequently linked to housing changes [43].

Pain management and constraint are essential to treat horses with colic. Moreover, regular and thorough monitoring of pain management is necessary to assess how well the treatment is working. In the present study, pain was treated with NSAIDs, namely, flunixin meglumine, which is the recommended medication for treating abdominal pain, according to the majority of previous findings. Flunixin meglumine is suggested to be the best analgesic for equine colic because of its better inhibitory power over visceral pain [44]. Among its many benefits are its ability to reduce inflammation and endotoxemia, as well as its ability to produce analgesia for 8–12 h by preventing prostaglandin synthesis, which is a key factor in the treatment of most basic medical cases of colic [45].

The availability of ketoprofen compared to flunixin, which is widely available, was the only factor that affected its use in the present study; its efficacy was not a factor. The choice of NSAIDs had no bearing on the results. According to another study [46], the choice of NSAIDs had some influence on pain signals but no significant impact on the clinical results in horses with strangulating small intestinal lesions. Ketoprofen is not frequently used to treat colic in horses, although it is an NSAID with analgesic properties comparable to flunixin meglumine [47]. Colic outcomes were positively impacted by the intubation of some horses. However, the nasogastric tube was primarily used in cases of colic impaction, consistent with the previous study by Guerra and Kilcoyne [48] suggesting that it was a means of decompressing and administering water and laxatives directly into the stomach. The contents of the colon can be softened and the impaction can be relieved by hydrating the colon or taking medicines such as magnesium sulfate, which promotes the flow of water into the lumen. Similarly, everything that promotes oral water intake and motility helps to resolve impaction [49]. As has been demonstrated in colic-stricken horses in this study, the administration of Vitamin B complex as a metabolic booster aids energy metabolism and marginally reduces lactic acid residue. In addition, Vitamin B complex may regulate large intestinal fermentation and boost appetite to improve endurance and catabolize energy during physical activity [50].

A limitation of this prospective study is the owner’s knowledge and experience with colic. Although abdominal pain is a general definition of colic, it must be supported by specific indications that can be recognized in a colic comparison diagnosis. There are a few possible causes of colic that need to be addressed to differentiate colic from illnesses that are not responsible for it but exhibit symptoms of abdominal pain. In a number of questions, the owner’s experience determines whether the colic is new or repeated. A portion of the data in this study has been gradually confirmed to the owners through direct investigations of horses to arrive at a precise diagnosis. It has also been verified in greater detail to obtain the same perspectives during the post-therapy monitoring phase. Further research on risk factors should take into account variables such as the number of horses cared for by the owners, their experience, and perspectives on preventive healthcare.

## Conclusion

This study concluded that the incidence of colic was reported in 187 cases (83.86%) in horses in Tuban, Indonesia, among 223 samples. Typical clinical parameters included HR >80 beats/min, abnormal breath rate, abnormal mucous membrane, moderate dehydration, profuse sweating, frequent urination, and elevated rectal temperature. Common signs of colic, such as abdominal pain, constipation, and anorexia, were also observed. Risk factors that contributed to colic were poor body condition score, wheat bran feeding, concentrate feeding >5 kg, less access to water, recurrence of colic, no deworming program, presence of gastrointestinal parasites, stressed physical activity, and summer season exposure. NSAID injections were first administered during the colic period, followed by fluid therapy, Vitamin B complex injections, and gastric intubation. These findings can be a recommendation for equine veterinarians to immediately perform therapeutic management according to ideal protocols and monitor factors that have the potential to contribute to the probability of colic.

## Authors’ Contributions

FF, MTEP, and HC: Conceptualized and designed the methodology and drafted the manuscript. DH, APW, and SC: Calculated and confirmed horse samples. AP, SK, and SC: Evaluated questionnaires and clinical parameters associated with colic in horses. FF, DH, APW, and MTEP: Investigated and diagnosed the horses referred with signs of colic. AP, SK, MTEP, and HC: Performed data curation and formal analysis. SK, SC, and FF: Edited the visualization and validation of tables. MTEP and HC: Revised and submitted the manuscript. All authors have read, reviewed, and approved the final manuscript.
